# Author Index for Volume 90

**DOI:** 10.1038/sj.bjc.6601977

**Published:** 2004-06-08

Aass, N 607, 2397

Abbou, M 888, 892

Abbruzzese, A 1710

Abedi, B 1402

Abeler, VM 678

Abeliovich, D 2002

Abnet, CC 1402

Aboagye, EO 2232

Abrosimov, A 2219

Abson, C 781

Adami, H-O 1378, 1386

Adamina, M 263

Adamo, V 2288

Adams, J 1109

Adib, TR 686

Ady, N 443

Afanasyeva, Y 153

Agarwal, R 810

Aghcheli, K 1402

Ahlgren, J 1740

Ahmad, SA 1620

Ahmedzai SH, 366

Airoldi, I 2210

Ajarim, DS 968

Ajayi, IO 638

Akagi, M 705

Akahira, J-i 2197

Akechi, T 787

Akhmedkhanov, A 153

Akiyama, S 1030

Alakhov, V 2085

Alberti, D 1498

Alberti, W 2305

Albetti, B 497

Albo, F 263

Albrecht, W 100

Ali, S 853

Allal, AS 471

Allasia, C 1216

Alldinger, I 1053

Allen, K 310, 314

Allen, NE 1392

Alleva, R 1644

Alliot, C 2050

Allis, CD 761

Allison, MC 1888

Alman, B 1443

Al-Malik, OA 968

Al-Shabanah, M 968

Altieri, A 1753

Altug, V 535

Ambagtsheer, G 1830

Ambaum, B 2268

Ambrosetti, A 353, 372

Amino, N 1600

Amlot, P 2402

Ammerpohl, O 1053

Andachi, H 672

Andera, L 1644

Anderson, E 41, 582

Anderson, J 1526

Anderson, NG 1295

Anderson, WP 822

Andrac, L 1216

Andreakos, E 1279

Andrew, A 1572

Andreyev, HJN 2278

Angen, M 2297

Angerson, WJ 1704

Anthoney, DA 601

Anti, M 882

Anttila, A 1756

Aoe, M 979

Aoki, M 2197

Aparicio, T 2051

Appleby, PN 118

Appleton, S 41

Aprikian, A 93

Archimbaud, A 503

Arends, MJ 1583

Ares, C 471

Arimochi, H 1672

Ariyoshi, Y 87

Armari, C 139

Armstrong, B 1382

Arndt, MAE 1863

Arslan, A 638

Arvelo, F 720

Ashley, S 1905

Ashman, JNE 900

Ashworth, A 1120

Aslam, M 874

Asosingh, K 1076

Astuti, D 515

Ateenyi-Agaba, C 1777

Aubert, J-P 657

Averbuch, S 566

Avigad, S 522

Avrahami, G 522

Awasthy, BS 1516

Azuma, Y 728

Babushkina, T 1833

Baccard, M 503

Baccarelli, A 497

Bachar, R 2194

Bader, SA 1972

Baguley, BC 906

Bajo, AM 245

Baker, C 1933

Bakker, RH 2073

Bakkus, M 1076

Baldwin, C 2278

Balibrea, JL 1983

Bamberg, M 2305

Bando, H 2338

Bara, J 720

Barber, MD 996, 1129

Barbera, S 2288

Bargo, S 2384

Barlesi, F 2097

Barlow, C 1318

Barni, S 1521

Barón, MG 1502, 1740

Barraclough, R 253, 1796

Barratt, A 321

Barrios, M 2050

Barry, JD 1888

Barthel, H 2232

Bartlett, JB 955

Bartolini, S 31, S 82

Basset-Seguin, N 503

Basso, O 1374

Basurto, C 36

Bates, DO 693

Batista, N 1740

Batlle, A 1660

Batra, SK 657

Batty, GD 1875

Bauer, J 1312

Baumann, F 911

Bauréus-Koch, C 48

Bearz, A 2288

Beck, J 985

Beckers, TL 2370

Begent, RHJ 2402

Beijnen, JH 2268

Bell, R 1133

Bellas, C 2145

Bellone, S 1814

Belluco, CL 1464

Benatti, P 882

Benavente, Y 2145

Benedetti, A 76

Bengtsson, NO 1942, 2344

Benito, M 1983

Bennett, P 1912

Benoit, G 55

Benson, JR 1349

Berardi, R 1521

Berberich, R 1989

Beretta, GD 1521

Berglund, 403

Berglund, G 122

Bergstrom, B 1133

Berner, Aa 449

Bernhard, J 1312

Bernstein, L 2138

Berrieman, HK 900

Berry, DP 1011

Bertani, MF 173

Berthou, C 139

Bertrand, G 503

Best, JM 1591

Beuthien-Baumann, B 620

Bhakta, D 160

Bhat-Nakshatri, P 853

Bilim, V 200

Binns, CW 1792

Bird, K 1583

Birembaut, P 1803

Birnie, E 383

Bisacci, C 36

Bishop, DT 2397

Bjørge, T 1876

Blackledge, G 566

Blackshaw, GRJC 1888

Blancato, J 1612

Bleumer, I 985

Blin, N 1594

Blomqvist, C 2344

Bloom, BS 26

Bobrow, LG 1349

Bocko, D 2042

Boddy, AV 60

Bodrogi, I 2397

Body, JJ 1133

Boecker, W 1422

Boersma, HH 745

Bogdanova, T 2219

Bohm, L 554

Boileau, C 1230

Boily, G 1606

Boivin, J-F 76

Boland, GP 423

Bolli, M 263

Bonel, HM 805

Bonfrer, JMG 2073

Bonnema, J 1144

Bonnier, P 1216

Bonomo, M 1312

Boocock, D 736

Boon, EMJ 224

Boonstra, R 2411

Booth, L 1926

Borghi, F 882

Borley, A 70

Borner, M 1312

Borràs, JM 1688

Børresen-Dale, A-L 678

Borsetti, M 693

Borsotti, P 2418

Bory, J-P 1803

Bosch, CJA 1543

Bosch, R 2145

Bosetti, C 1753

Boshoff, C 686

Bosma, AJ 1531

Botchway, SW 1450

Boulard, C 1230

Boulos, PB 2006

Bourmpoulia, D 686

Bouscarat, F 503

Boutard, P 139

Boven, E 1644

Bower, M 1526

Boyé, K 720

Braaten, T 1386

Brada, M 781

Bradbury, P 800

Bradley, C 2131

Bradshaw, TD 476

Brady, F 2232

Brady, G 1295

Brambilla, C 1222

Brambilla, E 1222

Brampton, M 2085

Brams, A 1230

Brancaccio, L 2288

Brandes, A 353

Brandt, B 1422

Brandt, BH 230

Bratti, C 146

Brauckhoff, M 911

Bray, CA 770

Bray, F 2157

Brazandeh, MH 1402

Breau, R 1093

Bréchot, J-M 2097

Brédart, A 106

Bredow, J 620

Brenner, B 1720

Brenner, H 1367

Brewster, AE 70

Brichon, PY 1222

Brind, J 2244

Brinkmann, AO 1041

Britton, PD 1349

Broggini, M 2418

Brown, G 2232

Brown, H 1486

Brown, S 1771

Brown, TLH 1457

Brown, WM 510

Brozzetti, S 2243

Brugieres, L 613

Brunetti, E 2243

Bruzzi, P 962

Bucana, C 705

Buccheri, G 2097

Buda, A 2112

Budach, V 2305

Bueno-de-Mesquita, BH 632

Bugat, R 343

Buijs-van der Woude, T 383

Bukowski, RM 1190

Bultz, BD 2297

Bundred, NJ 423, 1295

Bürger, H 1422

Burk, RD 146

Buskens, E 383

Butler, DM 1279

Butow, PN 321

Butt, IS 423

Buzelin, F 2364

Cabanes, A 2225

Cabras, MG 372

Calandri, C 82

Calderoni, A 353

Calista, D 497

Callahan, R 2384

Callegari, F 1100

Calvo, F 1727

Cameron, D 1933

Camirand, A 1825

Campbell, H 582

Campbell, J 41

Campbell, NC 1479, 1689

Campbell, RA 853

Campi, R 1374

Campion, L 189

Canaani, E 756

Cane, S 1814

Canna, K 1707

Cannon, MJ 1814

Canzonieri, V 2176

Cappello, F 938

Cappuzzo, F 31, 82

Carabantes, F 1740

Caraglia, M 1710

Cardarelli, N 1898

Carle, F 1898

Carlson, LE 2297

Carlsson, J 2344

Carlsson, P 1163

Carpentier, S 1216

Carraway III, KL 289

Carrozza, F 2288

Carruthers, SA 2080

Casas, A 1660

Cascinu, S 1521

Casetta, G 100

Casinello, J 1740

Cassidy, J 1479

Cassiman, J-J 1443

Cassinelli, G 1464

Castañón, C 1502

Castellanos, J 1740

Castells, A 206

Catalano, V 1521

Caudroy, S 1803

Cavazza, A 263

Cave, H 503

Cawkwell, L 900, 1976

Ceciarini, F 1710

Celerier, J 1059

Cellerino, R 1898

Ceresoli, GL 82

Cerny, T 1312

Cetnarskyj, R 582

Ceutto, GL 31, 1898

Chambers, AF 1877

Chan, H-L 173

Chan, PKS 1306

Chan, RC-F 1636

Chander, SK 932

Chang, B 510

Chang, GTG 1041

Chang, J-H 2149

Chang, S-Y 2186

Chang, Y-L 2378

Chanock, SJ 747

Chapuis Bernasconi, C 1059

Charles, P 2312

Chatelut, E 343

Chau, GY 2390

Chauveinc, L 443

Cheadle, JP 1591

Chen, BF 1995

Chen, H 510

Chen, J-S 1715

Chen, W-F 1636

Chen, Y-M 359

Chen, YD 2157

Cheng, A-L 1715

Cheng, D 944

Cheng, S-Y 430

Cheradame, S 526

Cherubini, R 36

Chester, KA 2402

Cheung, KL 590

Cheung, Y-B 1747

Chhatre, S 26

Chi, CW 2390

Chinchilli, VM 2244

Ching, L-M 906

Chipman, JK 1450

Chiu, J-H 2186

Cho, Y 1252

Choi, SH 1198

Choi, S-J 1329

Chong, G 483

Chou, S-C 728

Chouaïd, C 397

Choudhry, V 1863

Choudhury, A 657

Chow, K-C 2186

Chui, AY 558

Ciatto, S 393, 1784

Cid, O 492

Ciszak, L 2042

Civitelli, S 306

Claes, K 1244

Clahsen, PC 1543

Clarke, IA 955

Clarke, N 2142

Clarke, NW 542

Classe, J-M 189

Classen, J 2305

Clavel, C 1803

Clavel, J 139

Cleator, S 1120

Cocco, C 2210

Cockman, ME 1235

Coebergh, JWW 2332

Coelho, M 2032

Coene, I 1244

Cohen, B 1933

Coker, A 2171

Colditz, GA 299

Cole, M 60

Coleman, MP 1367, 2153

Coleman, N 1583

Collet, J-P 76

Collingridge, DR 2232

Collins, S 2142

Colombo, N 2112

Colomer, R 1688

Colozza, M 36

Colpaert, CG 1429

Combescure, C 397

Conroy, J 860

Constenla, M 1740

Conte, P 962

Conte, PF 31, 2112

Conti, E 2176

Contu, A 1898

Cook-Mozaffari, P 1019

Coombes, RC 1279

Cooney, KA 510

Cooper, N 1367

Correale, P 306, 1710

Corrias, MV 2210

Corvol, P 1059

Costantini, AS 1780

Cotter, TG 2025

Court, J 70

Courtenay-Luck, NS 1863

Coventry, BJ 939

Cowan, R 542, 2142

Cowell, JK 860

Cowen, ME 900

Cowie, S 483

Cox, B 2149

Crawford, SM 2131

Crellin, A 1343

Crichton, P 408

Crickx, B 503

Crinò, L 31, 36, 82

Croce, CM 756

Croce, M 2210

Crockford, G 2397

Cropp, C 2384

Crosby, TDL 70

Csizmadi, I 76

Cucherousset, J 1803

Cull, A 41, 310, 314

Cullum, J 2297

Cunningham, D 1190

Curran, S 1955

Cuzick, J 1109

da Silva, NF 515

Dachs, GU 1858

Dahm-Daphi, J 556, 1297

Dai, M 1777

Dal Maso, L 2176

Dales, JP 1216

D'Alessio, G 270

Dalgleish, AG 955

Dallol, A 515

Dalton, SO 1364

Daly, PA 2397

D’Angelo, A 1521

Danielsen, HE 678

Danson, S 2085

D’Antuono, T 2210

D’Aprile, M 1898

Darbha, R 1863

Date, H 979

Daugaard, G 1526, 2397

Daurès, J-P 397, 2097

Davies, MPA 182

Dawsey, SM 1402

Dawson, LA 1577

De Angelis, V 36

de Assis, S 2225

de Bock, GH 1144

de Bruijn, P 1508

de González AB 1787

de Haes, H 2123

de Heus, G 1508

de Jong, B 2411

de Jonge, MJA 1508

de Juan, C 1983

de Koning, HJ 383

de Krom, MCTFM 745

de Lumley, L 139

De Martino, A 306

De Mulder, P 985

de Pinieux, G 720

de Pouvourville, N 26

De Ruiter, A 1526

de Sanjosé, S 2145

De Stavola, B 2153

de Tejada, MRL 1502

de Ville de Goyet, J 1498

de Vries, EGE 2261, 2411

De Wever, O 1443

Dearnaley, D 2317

Decarli, A 1022

Degiuli, M 1727

Delecroix, V 189

Dell’Oro, S 353

Delvaux, N 106

DeMarchis, L 2384

Demidchik, EP 2219

den Bakker, MA 304

Denhardt, DT 463

Denis, MG 2364

Denkert, C 815

Dennison, AR 1011

Dent, J 2131

Denys, H 1443

Deonarain, MP 1863

Deporte, R 189

Descamps, V 503

Deschênes, J 483

deSouza, NM 2326

Devi-Ashok, T 1572

Dhara, S 1666

Dhillon, VS 874

Di Carlo, E 2210

Di Gaetano, S 270

Di Gregorio, C 882

Di Maio, M 2288

Di Salvo, E 2288

Di Vito, F 372

Diamandis, EP 167

Díaz-Rubio, E 1983

Dickson, RB 1612

Diel, IJ 1133

Dietel, M 815

Dietrich, D 1312

Dikomey, E 556

Dina, R 2326

D’Incalci, M 2418

Ding, I 430

Ding, SL 1995

Dinjens, WNM 888, 892

Dirix, LY 1429

Dite, GS 2149

Dittmar, T 230

DiVenosa, G 1660

Djuzenova, C 2356

Döbrössy, L 1887

Dobrowolski, F 1053

Dodson, A 182

Dodwell, D 1343, 2131

Domingo, A 2145

Dominguez, S 2051

Donati, S 31, 962

Dong, W 1777

Donnan, PT 1479

Donskov, F 626

Dooley, MJ 991

dos Santos Silva, I 160

Dosaka-Akita, H 1252

Dossi, R 2418

Doval, DC 1516

Dowdy, SF 230

Dowsett, M 2317

Dravet, F 189

Dredge, K 955

Drew, PJ 280

Driver, D 1169

Drize, O 773

Drye, ER 2006

Du Sart, D 483

Dubousset, J 613

Duffy, R 1479

Dufour, P 1190

Dulguerov, P 471

Dumez, H 2261

Duncan, ME 1019

Dundas, SR 1955

Dunlop, DJ 1704

Dunst, J 2305

Durocher, F 2002

Dusseldorp, E 1176

Duverger, V 2025

Dzik-Jurasz, ASK 781

Earl, HM 1349

Earl, J 408

Easton, D 2397

Ebbesen, P 1374

Ebert, W 2097

Eberwine, JH 1111

Edwards, GO 1450

Edwards, P 1888

Edwards, RT 1697, 1912

Eeles, RA 2002

Eggermont, AMM 1830

Ehninger, G 620

Einhorn, L 2397

Eisen, T 1905

El-Awady, RA 556

El-Helw, L 65

Elias, SG 115

Ellis, LM 705, 1620

Ellis, MJ 20

Emdin, S 1942

Engeland, A 1876

Engelholm, SA 377

Engels, EA 2181

Enger, SM 2138

England, K 2025

English, M 1498

Enomoto, N 216

Erichsen, HC 747

Erkelens, CAM 1654

Ernø, LE 1407

Escudero, P 1502

Eshleman, JR 1666

Eskens, FALM 1, 1508

Estève, J 1367

Etienne, M-C 526

Evans, PHR 560

Eves, P 1457

Ezenfis, J 2051

Ezzat, AA 968

Fadden MK 2171

Fahimi, S 1402

Faircloth, GT 2418

Falk, RT 146

Fallon, M 2245

Fan, F 705, 1620

Fannon, D 408

Farber, D 483

Farmer, PB 736, 1011

Farvacques, C 106

Fassina, G 833

Favini, E 1464

Fawole, A 638

Fearon, KCH 996, 1129

Fedeli, SL 1898

Feder, C 263

Federi, C 263

Federico, M 557

Feldmann, M 1279

Feliu, J 1502

Feltbower, RG 1882

Fennell, D 1318

Fenton, BM 430

Fernandez de Sevilla, A 2145

Fernet, M 866

Ferrarese, F 353

Ferraù, F 2288

Ferreri, AJM 353

Ferrini, S 2210

Ferry, D 2085

Ferry, DM 1084

Fiaschi, AI 306

Fidler, C 1211

Fields, ALA 1885

Fiorentini, G 1898

Fioritoni, G 372

Fischel, J-L 526

Fisher, M 1526

Fisker, R 626

Fizazi, K 55, 443

Flentje, M 2356

Fliessbach, K 1969

Floot, B 1259

Floriani, I 2112

Fondevila, C 206

Fontaine, J-J 720

Forman, D 652, 1343, 1926, 2397

Formento, J-L 526

Formento, P 526

Fornasarig, M 882

Forrest, LM 1704

Fortino, V 1293

Forward, DP 590

Fosså, SD 449, 607, 2397

Fossati, R 2112

Foster, CS 182

Foster, T 810

Foulkes, WD 483

Fourcade, J 343

Franc, B 1230

France, B 1912

France, M 1115

Franceschi, E 36

Franceschi, S 638, 1753, 1777, 2176

Francini, G 306, 1710

Franciosi, V 1521

Francis, RO 1666

Franco, E 93

Frankenburg, S 773

Frei, KA 805

French, D 2243

Frick, H 805

Friedlander, M 2397

Frontini, L 2288

Frost, G 2278

Fry, A 582

Frydecka, I 2042

Frydenberg, M 1374

Fujino, H 1672

Fujiwaki, R 1204

Fujiwara, T 979

Fujiyama, J 665

Fukuda, H 1660

Fukumoto, M 1204

Fukuoka, M 87, 2092

Fuloria, J 1516

Fumoleau, P 189

Funata, N 2338

Füssel, S 1034

Fuster, J 206

Gabbas, A 372

Gabriel, R 1803

Gaff, C 321

Gal, S 1211

Gallagher, C 1318

Gallamini, A 372

Galligioni, E 31

Gallo, C 2288

Gallo, E 372

Gandemer, V 139

Gandini, O 2243

Garbeglio, A 2176

Garcea, G 1011

Garcia, S 1216

García-Aranda, C 1983

Garcia-Corchón, C 1047

Garcia-Minaur, S 41

Garg, DK 1486

Gargantini, L 372

Gasc, J-M 1059

Gatta, A 833

Gattas, M 321

Gauthier, P 2032

Gazzard, B 1526

Geczi, L 2397

Geertsen, PF 1156

Gekeler, V 815

Gelderblom, H 304

Gendler, SJ 657

Genka, K 646

Gennari, A 31, 962

Gentle, D 515

Genuardi, M 882

Genze, F 455, 535

Gérard, B 503

Germà, JR 1688

Gertig, DM 2149

Gescher, AJ 736, 1011

Gesuita, R 1898

Gharbi, S 173

Ghaziani, R 1402

Giampieri, S 2203

Giardiello, FM 224

Giavazzi, R 2418

Giehl, K 455

Gierschik, P 455

Gietema, JA 2261

Giles, C 1169

Giles, GG 2149

Gillespie, K 1933

Gillessen, S 1312

Gillis, C 1920

Giorgi, F 1898

Giorgi, G 306, 1710

Glaholm, JG 2106

Glanzer, JG 1111

Glaser, AW 1882

Glaser, B 2002

Glasmacher, A 1969

Glimelius, B 403

Gnad, U 1893

Goedert, JJ 2145

Goffinet, M 343

Goh, C 1747

Gokden, M 1814

Goldacre, MJ 1019

Goldgar, D 483

Golomb, HM 1296

Gonen, M 1720

González-Quevedo, R 1983

Goodey, E 2297

Gopalan, D 1349

Gore, M 2245

Gore, ME 1156

Gori, S 36

Gorini, G 1780

Gorman, D 582

Goshen, Y 522

Gosseye, S 1498

Goto, K 787

Gottin, L 372

Gottschalk, AR 950

Gould, A 1933

Goumas, C 1382

Govi, S 353

Gozlan, Y 1833

Graeven, U 1190

Graf, W 403

Graham, J 601

Graham, T 483

Granath, F 1378

Grandchamp, B 503

Grau, JJ 206

Gray, J 1697, 1912

Graziano, F 1521

Green, J 1787

Greenbaum, L 1833

Greene, MH 2397

Greenman, J 900, 1976

Gregorc, V 82

Grenier, J 2097

Gridelli, C 2288

Grieu, F 419

Griffith, GL 1697, 1912

Griffith, M 1019

Grizzle, WE 1115

Grobbee, DE 115

Groen, HJM 2261

Groenewegen, G 2268

Groenewoud, JH 383

Gronlund, B 377

Groot, K 245

Grossman, L 497

Grossmann, ME 926

Grundy, R 515

Grünewald, K 1989

Grützmann, R 1053

Gschwend, A 1312

Gschwend, JE 535

Gu, C 436

Guarneri, V 962

Guarnieri, A 1710

Gubkin, A 353

Guerra, E 353

Guilford, P 2397

Guillou, PJ 1437

Guinebretiere, J-M 613

Gullane, PJ 560

Gullberg, B 122

Guo, D 2425

Guppy, AE 810

Gupta, S 1516

Gupta, SK 1516

Gutman, G 2194

Haan, E 321

Haba, R 1555

Habbema, JDF 1176

Habrand, J-L 613

Hackshaw, A 1302

Hagiwara, A 665, 1265

Hagmar, B 1407

Hake, SB 761

Halbert, G 2085, 2317

Hall, J 866

Hall, P 1378

Hamburger, T 773, 2002

Hamel, N 483

Han, C 1211

Hancock, B 1151

Hänel, A 620

Hänel, M 620

Hanley, JA 76

Hanna, E 1093

Hanselmann, S 1312

Hansen, HH 377

Hansson, J 403

Harada, M 1334

Harland, S 2317

Harland, SJ 1169

Harper, P 1190

Harper, SJ 693

Harris, AL 1211, 1235, 1429

Harrison, DJ 1972

Hartmann, JT 1893

Hartmann, O 613

Harvei, S 607

Hashida, H 1252

Hashimoto, S 1397

Hata, K 1204

Hatakeyama, K 2059

Haun, M 1989

Hauptmann, S 815

Hausheer, FH 1654

Hautmann, RE 535

Haward, R 1343

Hayakawa, N 135, 1397

Hayasizaki, Y 665

Haycock, JW 1457

Hayes, I 2025

Haynes, R 1169

Head, K 860

Heath, RM 1437

Heath, TM 1583

Heberer, M 263

Hedayati, M 497

Hedman, H 2425

Hehlmann, R 1893

Heidenreich, A 2397

Heimdal, K 2397

Heinzel, S 939

Helland, Å 678

Helms, MW 1422

Helsby, NA 1084

Hemminki, K 1765

Hémon, D 139

Henderson, S 686

Hendriks, JHCL 595

Hendry, JH 542

Heney, NM 578

Henley, J 2181

Henriksson, R 2425

Herben, VMM 2268

Herbert, A 1108

Hering, F 1312

Hernes, E 449

Herrero, R 146, 638

Herrington, CS 1949

Heun, R 1969

Hewitt, D 686

Hibi, K 1030

Hida, N 1334

Hida, Y 1252

Hilakivi-Clarke, L 2225

Hildesheim, A 146

Hill, RP 1842

Hinkula, M 1025

Hiorns, LR 476

Hiraki, Y 979

Hirao, S 1572

Hirao, T 1572

Hirata, K 1184

Hirose, S 348

Hirota, S 2059

Hirst, G 1450

Hisada, M 2181

Ho, WM 1306

Hochhaus, A 1893

Hockley, S 2397

Hoekstra-Weebers, JEHM 333

Hoey, RP 1295

Hofer, MD 455, 535

Hoff, PM 1190

Hofheinz, R-D 1893

Hofmann, R 985

Hofmann-Radvanyi, H 1230

Høgdall, C 377

Høgdall, EVS 377

Hogg, D 2397

Hokland, M 626

Hole, D 1920

Holland, R 595

Hollingsworth, MA 657

Hollingsworth, SJ 2006

Hollingworth, W 1349

Holloway, S 582

Holm, R 678

Holm, S 328

Holmberg, L 2167

Homs, MYV 2067

Honda, T 838, 2013

Hoogerbrugge, N 333

Hopper, JL 2149

Horenblas, S 100

Horgan, PG 1707

Hori, K 549

Horiguchi, S 2338

Horiike, A 1125

Horiuchi, C 348

Horn, L-C 911

Horn, O 408

Horwich, A 100, 294

Hoshiyama, Y 135

Hoskin, P 1151

Hoso, T 87

Hosoda, A 672

House, A 310, 314

Houssami, N 2118

Housset, B 397

Houterman, S 2332

Hövelmann, S 2370

Hoving, S 1830

Howitt, R 1538

Hsu, C 1715

Hsu, C-H 1715

Hu, B 430

Hua, L 1666

Huang, C 1555

Huang, G-W 2349

Huang, J-W 1809

Hubert, A 2002

Hubner, R 2245

Huddart, R 2397

Hughes, TP 12

Huibregtse, JM 194

Humphreys, AC 2131

Hunter, KW 752

Husain, SA 874

Husband, JE 2256

Hussain, SA 2106

Hutcheon, AW 601

Iacopetta, B 419

Iaffaioli, RV 2288

Ianniello, GP 2288

Ibrahim, EM 968

Ibrahim, K 1474

Iddon, J 1295

Iida, K 1204

Ikeda, H 844

Ikeda, T 216

Ikeda, Y 348

Ikemura, K 2062

Ikeya, T 1003

Ikezoe, T 2017

Ilc, K 526

Imai, K 844

Iniesta, P 1983

Inoue, S 2197

Iossa, A 1784

Ireson, CR 932

Irwig, L 2118

Isa, L 2288

Isaacs, SD 510

Isaacs, WB 510

Isacson, R 773

Ishida, T 844

Ishikawa, H 1003

Ishitoya, J 348

Islami, F 1402

Ismay, J 1318

Ito, H 672

Ito, K 1030, 2197

Ito, M 2219

Ito, Y 414, 1397

Itoh, K 1334, 1563

Itoi, T 200

Iwazaki, S 1003

Izumi, H 436

Izumi, N 216

Jackson, DG 1429

Jackson, L 590

Jadidizadeh, A 1443

James, ND 2106

James, NEA 1976

Jansen, S 2268

Janssen-Heijnen, MLG 2332

Janzer, R-C 1059

Jarman, M 2317

Jarry, A 2364

Jarvis, C 182

Jayadevappa, R 26

Jayne, DG 1437

Jenkinson, SR 253

Jenney, M 1498

Jensen, A 1687

Jewett, MAS 2397

Jézéquel, P 189

Ji, X 1863

Jian, L 1792

Jick, H 635

Jin, H 561

Joalland, M-P 189

Jodrell, DI 800

Joffe, JK 2131

Johansen, C 1364

Johansson, U 122

Johnson, L 2397

Johnson, M 1526

Johnson, PJ 1306

Johnson, R 2256

Johnsten, PG 282

Johnston, C 1343, 1926

Jones, A 1343

Jones, B 2326

Jones, DJL 736, 1011

Jones, DRB 1888

Jones, S 1591

Jones, W 601

Jones, WG 100

Jordan, VC 944

Joseph, D 419

Jou, Y-S 2378

Jourdan-Da Silva, N 139

Jowle, D 2085

Judde, J-G 720

Judson, I 2317

Juillerat-Jeanneret, L 1059

Julie, C 1230

Jung, YD 1620

Kaaks, R 115, 153

Kadouri, L 2002

Kaduri, L 773

Kahwa, B 1777

Kakinuma, S 1003

Kakudo, K 414

Kalaycio, M 281

Kalifa, C 613

Kalliomäki, T 1842

Kalman, S 100

Kalthoff, H 1053

Kamangar, F 1402

Kameyama, K 1555

Kanazawa, N 216

Kanda, T 2059

Kaneda, H 2092

Kanfer, EJ 1169

Kanzawa, T 1069

Karagas, MR 1572

Karam, G 2364

Karlsen, F 1407

Karoui, M 1230

Katagiri, K 1334

Katakami, N 87

Kataoka, A 2338

Kataoka, K 1672

Katayama, H 979

Katcoff, DJ 1833

Kato, I 153

Kato, K 1252

Katoh, H 1252

Katongole-Mbidde, E 1777

Katori, H 348

Katterle, Y 230

Kaufman, DS 578

Kauppila, A 1025

Kawaguchi, K 672

Kawaguchi, T 135, 1184

Kawahara, M 87

Kawai, S 348

Kawakami, T 1563

Kawata, S 838, 2013

Kawate, S 1003

Kayademir, T 1594

Kaye, JA 635

Kaye, SB 601

Kayl, AE 1691

Kehagioglou, P 70

Kehrer, DFS 1508

Keir, MJ 60

Kelland, LR 476

Keller, JJ 224

Kelly, CR 712

Kelsen, DP 1720

Kelsey, KT 1572

Kemp, EH 1457

Kerbusch, T 2268

Kerr, D 2085

Kersting, C 1422

Kersting, S 1053

Key, TJ 118, 1392

Khan, G 2225

Khatibian, M 1402

Khoo, K-S 1747

Kiehlmann, PA 1689

Kiessling, A 1034

Kievit, J 1144

Kikuchi, S 135

Kim, H-T 1329

Kim, MY 153

Kin, S 665

King, A 1457

King, AJ 853

King, DM 2256

Kingsmore, D 1920

Kinsey, SE 1882

Kishida, T 515

Kishimoto, Y 672

Kitajima, Y 1265

Kitaoka, S 672

Kiura, K 979

Klaren, A 1508

Kleekamp, N 975

Klimstra, DS 1720

Klockgether, T 1969

Kloover, JS 304

Klöppel, G 1053

Kneeshaw, PJ 280

Knight, A 1302

Knox, WF 423

Knüpfer, H 911

Knuth, A 985

Kobayashi, K 414

Kobayashi, M 128, 787

Köbel, M 815

Koch, R 1053

Kochanowska, I 2042

Koda, M 672

Kodama, T 1125

Koeffler, HP 2017

Koenig, KL 153

Koga, Y 1265

Kogner, P 515

Koh, W-P 1756

Köhler, U 911

Kohno, K 436

Koizumi, Y 1361

Kojima, M 1397

Kolch, W 283

Kölsch, H 1969

Kondo, K 1672

Kondo, M 1334

Kondo, S 1069, 1252

Kondo, T 135

Kondo, Y 1069

Kong, G 1198

Kong, Y 1443

Kooijman, R 1076

Koopmans, J 2297

Koppert, LB 888, 892

Korsching, E 1422

Korse, CM 2073

Kos, J 1961

Koskela, P 1025

Kosmaczewska, A 2042

Kote-Jarai, Z 2002

Kotzerke, J 620

Kovács, AF 1323

Kozuki, T 979

Kraft, H-G 1989

Kraus, I 1407

Krauss, J 1863

Kreil, S 1893

Kricker, A 1382

Kristeleit, H 955

Kristensen, GB 678

Kronblad, A 1942

Kropp, J 620

Kruit, W 985

Kudoh, S 87, 1184

Kuefer, R 535

Kühnel, G 620

Kuliczkowski, K 2042

Kuma, K 414, 1600

Kumar, DM 1474

Kumar, ID 1486

Kumle, M 1386

Kunitoh, H 1125

Kuniyoshi, H 646

Kunzi-Rapp, K 535

Kurata, T 2092

Kurebayashi, J 236

Kurokawa, T 1252

Kurosaki, M 216

Kusaba, T 1003

Kuwahara, O 646

Kuwahara, T 1672

Kyyrönen, P 1025

La Vecchia, C 1022, 1753, 2176

Labianca, R 1521

Laboisse, CL 2364

Lacapere, JJ 503

Laccetti, P 270

Laderoute, MP 278

Lakhani, SR 1538

Lalla, R 1457

Lam, EC 712

Lam, KC 1306

Lamagnére, JL 139

Lambert, S 1591

Lamberti, I 882

Lamers, C 985

Lamont, L 2297

Lancashire, RJ 1016

Landberg, G 1942

Landi, G 497

Landi, MT 497

Landucci, E 962

Lange, EM 510

Laniado, M 620

Lanternier, G 503

Lantuejoul, S 1222

Laplanche, A 55

Larsson, SC 2167

Latif, F 515

Laude, E 366

Lauge, A 866

Laukkanen, P 1025

Laurent-Puig, P 526

Laursen, TM 1364

Lavaut, MN 1216

Lawrie, LC 1955

Lazarev, A 1133

Lazarowicz, HP 1679

Lazzarino, M 372

Le, A-T 1443

Leach, MO 781

Leahy, MG 2397

Leake, R 1933

Lebbé, C 503

Ledermann, J 686

Lee, AH 1792

Lee, C-S 359

Lee, HC 2390

Lee, H-P 1760

Lee, JK 1198

Lee, KT 1198

Lee, Y-C 359, 2378

Lee, Y-J 1809

Lee, YW 1198

Leese, MP 932

Léger, F 343

Legrand, C 1543

Legrier, M-E 720

Lehtinen, M 1025

Leonard, CP 1666

Leonard, R 1933

Leong, S-S 1747

Lessing, JB 2194

Leu, FJ 1995

Levi, F 1022

Levick, JR 693

Lewis, IJ 1882

Lewis, JS 944

Lewis, MH 1949

Lewis, MP 822

Lewis, WG 1888

Leyland-Jones, B 476

Li, C-C 1715

Li, H 2181

Li, J 436

Li, LD 2157

Li, S 1093

Li, SH 2390

Li, W-Y 2186

Li, X 1765

Liao, DJ 1612

Liberzon, E 522

Lichinitzer, M 1133

Lim, W-T 1747

Lin, EY 2053

Lin, J 1285

Linch, D 1151

Lind MJ 900

Linja, MJ 1041

Linnebank, A 1969

Linnebank, M 1969

Liu, A 1612

Liu, C-J 1809

Liu, D 1555

Liu, E 1235

Liu, H-L 2349

Liu, H-T 1715

Liu, TY 2390

Liu, W 430, 705, 1620

Liubchenko, L 2397

LiVolsi, V 2219

LiVolsi, VA 1115

Ljungberg, 2425

Lo, S-S 1809

Lo, YMD 1211

Lobb, EA 321

Locatelli, M 353

Loehrer, PJ 2181

Lohynska, R 2397

Lokich, J 1190

Lombardo, L 82

Looijenga, LHJ 2397

Loos, WJ 343

López-Gómez, L 1502

López-Picazo, JM 1047

Lorenzato, M 1803

Lorenzi, M 306

Lorimier, P 1222

Losi, L 882

Lotem, M 773

Louwman, WJ 2332

Lozano, MD 1047

Lu, W-Q 2349

Lu, Y-S 1715

Lubsen, MAC 1492

Lucci-Cordisco, E 882

Lucentini, A 31

Lui, W-Y 1809, 2390

Luiten, E 2123

Lund, E 1386

Lundqvist, H 2344

Lunec, J 1679

Luoto, R 1756

Lushnikov, E 2219

Luthra, SK 2232

Lüttges, J 1053

Lutz, P 139

Lygoe, KA 822

Lynge, E 1687

Lyons, S 1259

Mabed, M 65

MacDonald, A 1976

MacKie, RM 770

Maclennan, K 1151

MacNamara, E 483

MacNeil, S 1457

MacRae, JH 2297

MacRobert, AJ 1660

Madajewicz, S 1190

Madsen, HHT 626

Maeda, Y 1334

Maerki, HP 1059

Magnusson, C 1378

Maguire, P 310, 314

Maher, ER 515

Mahmud, S 93

Mahon, MM 2326

Mahteme, H 403

Maioli, E 1293

Maitland, NJ 1577

Maitra, A 1666

Mala, C 985

Malafosse, R 1230

Malaguti, C 1100

Malekzadeh, R 1402

Mali, WPThM 383, 595

Malik, Z 1847

Malvezzi, M 1022

Manenti, L 2418

Mangioni, C 2112

Mangtani, P 160

Mann, JI 118

Mansfield, PF 705

Mansutti, M 31

Manzano, RG 1047

Manzione, L 2288

Mao, Y 1138

Marchal, S 106

Marchese, R 2243

Marcus, VA 483

Marcussen, N 626

Maréchal, JM 100

Mareel, M 1443

Margison, GP 542

Margison, J 2085

Margueritte, G 139

Marinelli, B 497

Maring, JG 2261

Marino, M 833

Marjani, HA 1402

Maroun, JA 1190

Marrazzo, A 1414

Marsh, S 8

Marshall, JF 822

Marshall, JL 1190

Marshall, MS 853

Marshall, V 2145

Marsili, S 306, 1710

Marti, WR 263

Martignoni, G 1521

Martin, M 2297

Martín, MS 1502

Martín-Algarra, S 1047

Martínez, MP 1502

Martinsson, T 515

Marugame, T 646

Marzocca, G 306

Mason, M 2317

Masuya, D 1555

Mathieu, M-C 443

Mathijssen, RHJ 343, 1508

Mathôt, RAA 2268

Matichard, E 503

Matsuda, H 348

Matsueda, S 1563

Matsui, K 87

Matsuura, N 414

Matsuzuka, F 414

Mattisson, I 122

Maughan, TS 70

Maxwell, P 1235

Mayer, A 2402

Mazo, A 756

Mazzoni, F 31

McArdle, CS 1704, 1707

McCallum, M 1933

McCarty, MF 705, 1620

McCormack, VA 160

McCredie, MRE 2149

McGough, C 2278

McIndoe, A 2326

McKean, MJ 601

McKee, RF 1707

McKiernan, P 1498

McKinney, PA 1882

McLeod, HL 8

McMaster, M 2397

McMichael, AJ 160

McMillan, DC 1129, 1704, 1707

McNeish, IA 1169

McNicol, A-M 1707

McQuaid, D 860

McVety, S 483

Meazza, R 2210

Méchinaud, F 139

Meijer, CJLM 638

Meiser, B 321

Meisner, C 2305

Melchior, S 985

Meldrum, RA 1450

Mellon, JK 1679

Menestrina, F 372

Menke, A 455

Menu, E 1076

Menzel, C 1551

Menzel, H-J 1989

Merat, S 1402

Mercadante, S 2049

Merims, S 773

Meschino, W 483

Messiaen, L 1244

Messinese, S 306, 1710

Metges, JP 206

Meyers, CA 1691

Meziani, R 503

Mhawech, P 471

Miccinesi, G 1780

Michael, A 955

Michaelson, MD 578

Michalaki, V 2312

Micheli, L 1710

Mickisch, G 100

Mikami, Y 348

Mikeljevic, JS 1343

Milano, G 526

Millar, D 1479

Miller, E 1474

Millis, RR 1538

Millot, F 139

Millour, M 189

Minafra, S 1414

Minard-Colin, V 613

Minghetti, P 497

Minton, NP 2402

Mirebeau, D 503

Mita, H 844

Mitchell, EP 1190

Mitry, E 1367, 2051

Miura, Y 135

Miya, A 414

Miyagawa, K 665

Miyamoto, M 1252

Miyaoka, H 787

Miyasaka, Y 216

Miyauchi, A 414, 1600

Miyazaki, K 1204, 1265

Miyoshi, A 1265

Miyoshi, E 414

Miyoshi, H 701

Miyoshi, T 1672

Mizoue, T 135

Mochimatu, I 348

Molden, T 1407

Molenaar, S 2123

Molinier, L 397

Molinier, O 2097

Molino, A 31

Monden, Y 1672

Moniaux, N 657

Montella, M 2176

Montgomery, PQ 560

Montironi, R 1898

Montuenga, LM 1047

Moog, U 333

Mooij, TM 1492

Moore, J 1211

Moore, P 182

Morán, A 1983, 2142

Morant, R 1312

Morat, L 443, 1222

Moretti, S 1644

Mori, T 756

Morimoto, Y 844

Morishita, Y 1003

Moriya, T 2197

Moro-Sibilot, D 1222, 2097

Morra, E 372

Morris, JS 1392

Morris, LS 1583

Morrison, E 1437

Morriss, J 1138

Mortensen, PB 1364

Mortimer, PS 693

Mosconi, AM 36

Moses, AWG 996

Moskaluk, CA 1115

Motoyama, T 838, 2013

Moullan, N 866

Moye, VE 1796

Mugino, H 2062

Mühl, B 2356

Mukohara, T 1184

Mulder, J 2123

Mulders, PFA 985

Muleti, A 2243

Muley, T 2097

Mullee, MA 2153

Muller, GW 955

Muñoz, N 638

Munro, A 1479

Munzer, M 139

Murai, M 844

Murawaki, Y 672

Murias, A 1740

Murphy, A-M 2025

Murphy, FJ 2025

Murphy, PS 781

Murray, GI 1955

Muta, M 2338

Muti, C 1230

Nakagawa, K 87, 194, 2092

Nakagawa, S 194

Nakajima, M 1873

Nakajima, T 1003

Nakamura, S 1003, 2062

Nakamura, T 756

Nakano, T 87

Nakao, A 1030

Nakaoka, Y 1184

Nakase, Y 665

Nakashima, S 665

Nakashima, T 1555

Nakaya, N 1361

Nakayama, H 1030

Nakayama, K 1204

Nakayama, T 646

Nakshatri, H 853

Narod, S 483

Nasseri-Moghaddam, S 1402

Natsukawa, S 646

Naumann, R 620

Navarro-Teulon, I 2032

Nazeyrollas, P 1803

Negoro, S-I 87

Negri, E 1022, 2176

Negrier, S 1156

Nelson, HH 1572

Nelson, M 1526

Nelstrop, AE 810

Nencini, C 1710, 1710

Neri, A 1710

Nesland, JM 449

Neuzil, J 1644

Newlands, ES 1169

Newman, SP 932

Newton, DL 1863

Newton, TR 853

Ng, G-Y 1747

Nguyen, G 1059

Ngwenyama, RA 1392

Nicholson, S 955

Niggemann, B 230

Niikura, H 2197

Nishiwaki, Y 787

Nishizawa, N 646

Nishizuka, S 838, 2013

Nitti, D 833

Njor, SH 1687

Nogami, T 2092

Nokihara, H 1125

Nordgren, H 2344

Nordlinger, B 1230

Nortilli, R 1898

Norton, A 1905

Nou, JM 1803

Nouraie, M 1402

Nowak, NJ 860

Nusgens, B 1443

Nutley, B 2317

Nutt, JE 1679

Nyberg, G 48

Nygren, P 403

Nystrom, M 1318

Nystrom, ML 822

Oblak, I 1961

O’Brien, MER 1905

O’Brien, TJ 1814

Ockert, D 1053

Odefrey, F 483

O’Donnell, A 2317

Oertli, D 263

Oesterling, C 1318

Offerhaus, GJA 224

Ohashi, A 2059

Ohe, Y 1125

Ohnishi, Y 1672

Ohno, S 2338

Ohno, Y 135

Ohwada, S 1003

Ojemakinde, K 638

Okamura, K 2197

Okazaki, Y 665

Okubo, S 236

Okushiba, S 1252

Oladepo, O 638

Olah, E 2397

O’Leary, R 1437

Oliver, DT 2397

Oliver, T 1526

Olivier, RI 1492

Ollivaud, L 503

Ollivier, L 2256

Olsen, AH 1687

Olsen, J 1374

Olsson, H 122

Omigbodun, AA 638

O'Neill, E 283

O'Neill, PA 182

Ong, J 1526

Onland-Moret, NC 115

Ono, M 348

Oort, F 2123

Oosterwijk, E 985

Oosterwijk, JC 333

Oppitz, U 2356

Orlandi, E 372

Orlandini, C 31, 962

Orlando, A 693

Ormiston, WJ 2397

Öry, FG 2135

Osaki, T 436

Oshima, M 701

Osman, S 2232

Ostenstad, B 2344

Otnes, B 449

Otsuki, T 236

Otte J-B, 1498

Ottó, S 1887

Oya, R 2062

Oyama, T 436

Ozols, RF 559

Paavonen, J 1025

Paci, E 393, 1780, 1784

Packeisen, J 1422

Padovan, E 263

Paepe, AD 1244

Paesmans, M 2097

Påhlman, L 403

Paik, SS 1198

Pal, T 483

Palacín, A 206

Palestro, M 372

Palmer, PA 1508

Palmieri, M 1814

Pals, ST 224

Pandha, H 955

Pang, X-W 1636

Paoni, SF 430

Pappo, I 773

Parish, D 932

Park, YH 1329

Parker, AR 1666

Parkin, DM 2157

Parry, A 60

Parziale, AP 1898

Pasini, F 353, 372

Patel, NM 853

Patmore, HS 1976

Patterson, AV 1084

Paul, J 601

Paulli, M 372

Pautard, B 139

Pauzner, D 2194

Paxton, J 1933

Peake, DR 2106

Pearson, ADJ 60

Pecherstorfer, M 1133

Pecorelli, S 1814

Pedroni, M 882

Peeters, PHM 115, 595

Peintinger, F 1551

Pèlegrin, A 2032

Pelletier, G 2297

Pels, H 1969

Pelucchi, C 1022, 1753

Penna, C 1230

Pera, M 206

Perel, Y 139

Perera, S 932

Peretz, T 773, 2002

Perez-Manga, G 1190, 1740

Pericay, C 1502

Perng, R-P 359

Perona, R 573

Perotti, C 1660

Perren, TJ 2131

Perrett, C 686

Perrone, F 2288

Perschky, L 70

Persson, BRR 48

Peterse, HL 1531

Peterse, JL 595

Petrangolini, G 1464

Petrioli, R 306, 1710

Pettengell, R 1151

Phillips, K-A 2397

Piazza, E 2288

Piazza, T 2210

Piccoli, R 270

Piccolomini, A 1710

Picton, S 1882

Piga, A 1898

Pijnappel, RM 383, 595

Pilarsky, C 1053

Pinder, SE 1538

Pisani, P 652, 1343

Pistoia, V 2210

Pivnik, A 353

Pizzolo, G 372

Pizzuti, M 372

Planting, AST 1508

Plouvier, E 139

Polette, M 1803

Poli, M 2418

Polinder, S 2067

Pollak, M 1825

Pollard, JW 2053

Polychronis, A 955

Polymeris, M-E 167

Ponti, A 1727

Ponti, G 882

Ponz de Leon, M 882

Popalis, C 167

Poppe, B 1244

Poppema, S 2411

Porro, M 2261

Porteous, M 41, 582

Porter, JF 463

Postma, K 314

Potter, BVL 932

Potter, GA 736

Poupon, M-F 720

Pour, PM 657

Pourshams, A 1402

Powell, SN 1297

Powles, T 1526

Pozzessere, D 306

Prapotnich, D 443

Prasad, R 423

Pratesi, G 1464

Preiss, R 911

Preston, T 996, 1129

Prete, SD 1710

Price, L 60

Price, PM 2232

Priest, K 1905

Procopio, A 1644

Prokop, E 1551

Pruvot, F-R 1230

Pucci-Minafra, I 1414

Pugnière, M 2032

Pujol, J-L 2097

Pukkala, E 1025, 1756

Pullen, SM 1084

Purdie, C 1933

Purdy, D 2402

Purohit, A 932

Purushotham, AD 1349

Puy, H 1230

Qian, D 1285

Qualman, SJ 1115

Quereux, C 1803

Quero, C 1047

Quinn, MJ 1367

Quoix, E 2097

Rachet, B 1367

Radvanyi, F 1230

Rahal, MM 968

Raitanen, J 1756

Raja, MA 968

Raleigh, JA 728

Ramakrishnan, S 1627

Ramani, P 1498

Ramirez, A 310, 314

Ramirez, NC 1115

Ramsey, D 1594

Ramuz, O 1216

Ranson, M 2085, 2250

Rao, MS 558

Rapley, EA 2397

Ratanatharathorn, V 1093

Ratcliffe, PJ 1235

Ravaioli, A 2112

Ray, RM 2244

Raynaud, F 2317

Razavi, D 106

Reed, MJ 932

Reed, MWR 1486

Regazzi, M 353

Regueiro, P 1740

Reiß A 620

Reinmuth, N 705, 1620

Reitsamer, R 1551

Renaudin, K 2364

Reni, M 353

Reschner, A 263

Rettenbacher, L 1551

Reznek, R 2256

Rialland, X 139

Ribatti, D 2418

Ricci, S 962

Richard, S 2397

Richards, J 1863

Richards, M 1882

Richardson, AK 2149

Ricolleau, G 189

Rieber, EP 1034

Rieger, MA 1034

Riga, M 1367

Rigby, H 693

Rinaldi, S 115, 153

Rippo, MR 1644

Rischin, D 991

Ritchie, A 794

Ritchie, LD 1479

Rittling, SR 1877

Roach III, M 950

Robanus-Maandag, EC 1543

Robbiati, SF 2288

Robbins, A 2317

Robert, A 139

Roberts, JT 601

Robertson, A 366, 1933

Robertson, JFR 590

Robertson, R 1479

Robinson, J 2297

Robreau, A-M 1230

Rocca, GL 1414

Rochlitz, C 1312

Rodrigues, RF 492

Rodriguez, AC 146

Rodríguez-García, JM 1502

Rodwell, S 2131

Rognone, A 353

Roman, JJ 1814

Romanengo, M 2153

Roncucci, L 882

Rondini, M 962

Ronzitti, G 1100

Rookus, MA 1492

Roppongi, T 1003

Roque, L 492

Roquet, F 2032

Rosai, J 2219

Rosa-Santos, J 492

Rosell, R 1688

Rosetti, F 2288

Rosing, H 2268

Ross, DM 12

Ross, JA 1129

Ross, PJ 1905

Rossel, P 328

Rossi, G 372

Rossi, R 2112

Rossini, GP 1100

Rougier, P 1190, 1230

Rowland, IJ 781

Royston, P 794

Rozovskaia, T 756

Rudland, PS 253, 1796

Ruiz, A 1740

Rulli, A 36

Rumping, G 1259

Ruparelia, KC 736

Rush, R 41, 310, 314, 582

Rushbrook, SM 1583

Rustin, G 2326

Rustin, GJS 810, 1169

Rutgers, E 2123

Rutgers, EJ 1531

Ryan, A 705

Rybak, SM 1863

Rydén, L 1942

Ryder, K 1538

Rydh, B 1378

Ryoo, B-Y 1329

Sabatier, L 443, 1222

Sabatino, M 306

Saeger, HD 1053

Saïag, P 503

Saijo, N 1125

Saikali, Z 1606

Sainsbury, R 1733

Saito, H 87, 1873

Saito, S 549

Saji, S 2338

Sakakura, C 665

Sakamoto, N 216

Sakata, K 135

Sakuma, Y 348

Sale, S 736

Salford, LG 48

Salgado, R 1429

Salud, A 1502

Salvadori, B 31, 962

Salvagni, S 1521

Salway, F 1279

Sampson, JR 1591

Sánchez, JJ 1502

Sánchez-Pérez, I 573

Sánchez-Pernaute, A 1983

Sandblom, G 1163

Sanders, PM 1274

Sanderson, C 1295

Sanderson, M 2171

Sangar, VK 542

Sanjoaquin, MA 118

Santin, AD 1814

Santoro, M 270

Saramäki, OR 1041

Sarmanova, J 1989

Sasaki, N 414

Sasaki, S 128, 787

Sasako, M 1727

Sasano, H 2197

Sasatomi, T 1334

Sasazuki, S 128

Sasiadek, MM 1594

Sasieni, P 1109, 1784

Sato, C 216

Sato, K 1265, 1334

Sato, M 1334

Sato, N 844

Sato, Y 1334, 1563

Satoh, A 844

Satoh, T 2092

Sauerbrei, W 794

Saussele, S 1893

Savelyich, BSP 1474

Savinainen, KJ 1041

Sawada, N 1672

Scartozzi, M 1521

Schackert, HK 1053

Schakowski, R 2356

Schally, AV 245

Schejter, E 2194

Schellens, JHM 2268

Schernhammer, ES 941

Scheungraber, C 975

Schiffman, M 146

Schilsky, RL 1190

Schlegel, U 1969

Schmid, H-P 1312

Schmidberger, H 2305

Schmidt, M 815, 2370

Schmidt, R 911

Schmidt, S 1969

Schmidt-Wolf, IGH 1969

Schmied, BM 657

Schmiegel, W 1190

Schmitz, M 1034

Schned, A 1572

Schneider, A 975

Schneider, D 1771

Schoelmerich, J 1190

Schönfelder, M 911

Schulmeister, K 941

Schulze, J 620

Schumacher, R 263

Schutte, M 888

Schwartz, DI 1833

Schwartz, LH 2256

Schwartz, W 1687

Sciot, R 1443

Scott, IS 1583

Secchi, S 372

Seckl, MJ 810, 1169

Sekhon, JS 1516

Sekine, I 1125

Selby, P 1926

Semenciw, R 1138

Semnani, S 1402

Senba, H 87

Sennfält, K 1163

Serretta, V 100

Servitje, O 2145

Seydel, C 408

Shaaban, AM 182

Shah, MA 1720

Shamaa, S 65

Shamash, J 1526

Sharif, K 1498

Sharma, RA 1011

Sharma, SK 2402

Sharpe, M 310, 314

Shaughnessy Jr, J 1814

Sheehan, D 2025

Shen, CY 1995

Shen, S 463

Shen, Y-C 1715

Sheng, ZM 2384

Sherman, ME 146

Sheu, LF 1995

Shia, J 1720

Shichijo, S 1563

Shields, JD 693

Shields, TS 146

Shih, J-F 359

Shih, J-Y 2378

Shiloni, E 773

Shimizu, N 979

Shimomura, K 665

Shinohara, T 1334

Shiota, G 672

Shipley, WU 578

Shirai, Y 2059

Shoemaker, WJ 2225

Shomura, H 1334, 1563

Shore, RE 153

Shukla, VK 1516

Sibson, DR 182

Sidoni, A 36

Sidorov, Yu 2219

Siersema, PD 2067

Silva, RR 1521

Simpson, JSA 2297

Singh, B 1612

Singh, R 1011

Singh, S 991

Sinnett, D 1606

Sipos, B 1053

Sjöström, J 2344

Skelton, LA 476

Skomedal, H 678, 1407

Slachmuylder, J-L 106

Slater, C 996

Smet, A 1777

Šmid, L 1961

Smigiel, R 1594

Smith, AC 932

Smith, HJ 1850

Smith, IE 1905

Smith, JS 638

Smith, P 1151

Smith, S 1479

Smith, ST 756

Snijders, PJF 638

Soares, J 492

Sobrero, A 1190

Sobue, T 646

Söderberg, M 1740

Sondak, VK 1285

Sonoo, H 236

Soosaipillai, A 167

Sorahan, T 1016

Sorbris, R 968

Sørensen, JB 328

Soria, J-C 443, 1047, 1222

Sorrentino, R 270

Sotiriou, C 1235

Sotoudeh, M 1402

Soucek, P 1989

Souchon, R 2305

Soufir, N 503

Southern, SA 1949

Southey, MC 2149

Soutter, WP 2326

Sowerbutts, A-M 2142

Spagnoli, GC 263

Spalletti-Cernia, D 270

Sparreboom, A 343, 1508

Spatafora, M 2288

Speca, M 2297

Speight, PM 822

Spence, RAJ 282

Spencer, J 2256

Spicer, J 1151

Spina, M 353

Sprangers, M 2123

Spreafico, A 82

Stachura, S 1285

Stafford, ND 1976

Stalmeier, PFM 333

Stamerra, O 55

Standop, J 657

Stark, B 522

Staroz, F 1230

Stebbing, J 1526

Steel, M 582, 1933

Stein, CJ 299

Steiner, RA 805

Stembalska–Kozlowska, A 1594

Stendahl, M 1942

Stenning, SP 1176

Stessels, F 1429

Stevanovic, S 1034

Stevens, RG 2225

Stevenson, L 1689

Steward, WP 736, 1011

Stewart, FA 1259

Stewart, H 1933

Steyerberg, EW 1176, 2067

Stiggelbout, AM 1144

Stirling, DI 955

Stocken, DD 2106

Stoeltzing, O 705, 1620

Stoll, H 2397

Stoppa-Lyonnet, D 866

Storey, A 2203

Stratton, MR 2397

Strojan, P 1961

Strong, V 310, 314

Struewing, JP 497

Suen, J 1093

Sugio, K 436

Sugiura, T 87

Sullivan, F 1479

Sumi, K 1265

Sunagawa, K 646

Sundar, S 1474

Supino, R 1464

Suthers, G 321

Suzuki, H 844

Suzuki, K 1397

Suzuki, S 787

Suzuki, T 646, 2197

Suzuki, Y 194, 1361, 1361

Svetic, B 1961

Swart, M 2268

Sweeney, C 289

Sweetenham, J 1302

Sweetland, S 1787

Swerdlow, AJ 294

Sylvester, R 100

Symonds, RP 1474

Syrigos, K 2312

Szepeshazi, K 245

Szturmowicz, M 2097

Taal, BG 2073

Tabata, M 979

Taghavi, N 1402

Taguchi, H 2017

Taguchi, T 348

Tai, S-K 2186

Takada, M 2338

Takada, Y 87

Takagi, S 2062

Takagi, T 665

Takahashi, K 200

Takahashi, M 1334

Takahashi, T 1003

Takahashi, Y 1672

Takamura, Y 414, 844

Takano, T 1600

Takebayashi, Y 1204

Takeda, A 705

Takeda, H 701

Takemoto, M 979

Takemura, M 665

Takenawa, H 216

Takenoyama, M 436

Taketani, Y 194

Taketo, MM 701

Takeuchi, H 1069

Takizawa, S 194

Talamini, R 1753, 2176

Talbot, DC 800

Tamai, Y 701

Tamakoshi, A 135, 1397

Tamakoshi, K 1397

Tamberi, S 82

Tammela, TLJ 1041

Tamura, G 838, 2013

Tamura, K 2092

Tamura, T 1125

Tan, E-H 1747

Tanaka, K 236

Tanaka, N 979

Tang, H-J 1285

Tangerman, A 632

Tani, F 306

Tanigaki, Y 348

Taniguchi, K 787

Tanimoto, M 979

Tanzini, G 306

Taormina, P 1414

Taraboletti, G 2418

Taranger-Charpin, C 1216

Tarella, C 372

Tavani, A 2176

Taylor, M 1211

Taylor, PR 1402

Tecchio, C 372

Tejpar, S 1443

Temme, A 1034

ten Bokkel Huinink, WW 2268

ten Hagen, TLM 1830

Terashima, M 838

Terrier-Lacombe, MJ 55

Tew, KD 2411

Thalib L 1378

Tharakan, G 2402

Theodore, Ch 55

Thiery, JP 1230

Thiffault, I 483

Thomas, DB 2244

Thomas, GA 2219

Thomas, GJ 822

Thomas, JO 638

Thomasson, M 2425

Thorogood, M 118

Tian, E 1814

Tilanus, HW 888, 892

Tillotson, L 2297

Timmer-Bosscha, H 2411

Timms, JF 173

Tisdale, MJ 1274, 1850

Tjulandin, SA 2397

Todeschini, G 372

Todo, S 1334, 1563

Toi, M 2338

Tokino, T 844

Tokudome, S 1397

Tokui, N 135

Toledo, G 1047

Tolner, B 2402

Tomasetti, M 1644

Tomita, F 200

Tommasino, M 1777

Tomoda, C 414

Tonato, M 36

Toniolo, P 153

Torre, W 1047

Torres, A 1983

Torri, V 2112

Tortoreto, M 1464

Torun, E 1654

Toschi, L 82

Tou, SIH 2006

Tourani, JM 1156

Toyoshima, H 135, 1397

Toyota, M 844

Tozer, GM 1858

Tresallet, C 1230

Tretli, S 607, 1876

Tripathy, D 1133

Tronko, MD 2219

Tropé, CG 678

Troy, G 366

Tsai, C-M 359

Tschanz, E 471

Tseng, T 497

Tsubono, Y 1361

Tsubura, E 646

Tsugane, S 128, 787

Tsuji, I 1361

Tsukuda, M 348

Tsutsumi, A 672

Tsyb, AF 2219

Tucker, K 321, 2397

Tulbah, AM 968

Tulloh, A 314

Tumolo, S 31

Tupper, J 1858

Turnbull, LW 280

Turner, AJ 1577

Turner, J 1912

Uchitomi, Y 787

Ueno, M 1555

Ueno, T 2338

Ueoka, H 979

Ugnat, AM 1138

Ullrich, S 985

Ulrich, AB 657

Une, Y 1334

Uruno, T 414

Usmani, BA 1577

Valduga, F 31

Valente, M 833

Vallette, G 2364

van Beurden, M 1492

van Blitterswijk, WJ 917

Van Camp, B 1076

Van Cutsem, E 1190

van Daal, WAJ 333

Van Dam, K 1443

van de Velde, CJH 1144, 1543

van de Vijver, MJ 1492, 1543

van de Wetering, M 892

van den Akker-van Marle, ME 383

van den Berg, A 2411

van den Berg, D 1760

van den Broek, LJCM 1543

van den Donk, M 595

Van den Eynden, G 1429

Van den Heuvel, E 1429

van den Ouweland, AMW 892

Van der Auwera, I 1429

van der Hage, JA 1543

van der Kolk, DM 2411

van der Kooy, K 2135

van der Neut, R 224

van der Sangen, MJC 2332

van der Velden, AW 892

van der Vijgh, WJF 1654

van der Wall, E 1422

van Diest, PJ 1422

van Dijk, MR 1176

van Echten-Arends, J 2411

van Faassen, A 632

van Leeuwen, FE 2135

Van Marck, EA 1429

van Meerbeeck, JP 304

van Noord, PAH 115

van Oosterom, AT 2261

van Peppen, AM 2135

Van Poppel, H 100

van Rijswijk, REN 745

van Roosmalen, MS 333

van Tiel, ST 1830

Van Valckenborgh, E 1076

van Vliet, C 2118

Vanderkerken, K 1076

Vannier, JP 139

van’t Veer, LJ 1492, 1531

Varenhorst, E 1163

Varga, Z 985

Varia, MA 728

Varner, J 561

Vassal, G 613

Veal, GJ 60

Vecchio, G 270

Vejborg, I 1687

Veldhorst-Janssen, NML 745

Veldman, RJ 917

Velikova, G 1926

Verdan, C 471

Verduijn, P 1531

Vergnenègre, A 397

Verhaeghe, T 1508

Verheij, C 1508

Verheij, CDGW 2332

Verheij, M 917

Verhoef, LCG 333

Vermeulen, PB 1429

Verschoyle, RD 736

Verschraagen, M 1644

Verweij, J 343, 1508

Veyrenc, S 1222

Vicent, S 1047

Viel, A 882

Villa, E 82, 353

Villadsen, E 1687

Villman, K 1740, 2344

Visakorpi, T 1041

Visioli, C 393

Visioli, CB 1784

Visser, O 2135

Vitolo, U 372

Viviers, L 781

Voest, EE 2268

Volant, A 206

Volkes, EE 1296

von der Maase, H 626, 1156

Vora, V 366

Voss, R 1422

Vowler, SL 1583, 2219

Vreugdenhil, G 2332

Vu, BK 1863

Vuolo, G 1710

Wabinga, H 1777

Wachters FM 2261

Wainscoat, JS 1211

Wakai, K 1397

Wakai, T 2059

Waki, T 838, 2013

Wall, L 800

Wallace, AM 1129

Wallace, DMA 2106

Wallington, MM 2080

Walsh, PC 510

Walt, H 805

Wang, L-S 2186

Wang, M 2225

Wang, M-H 926

Wang, W 2349

Wang, Y 678

Wang, Y-D 860, 1636

Wang, Y-F 2186

Warren, RML 1349

Warren, W 2397

Wartenberg, D 1771

Washington, MK 1115

Watanabe, M 216

Watanabe, Y 1397

Waterfield, MD 173

Waters, JS 1905

Waterston, AM 1279

Watson, M 41

Watters, AK 483

Waxman, J 2312

Weber, BL 2397

Weber, W 2397

Wedrén, S 1378

Wefel, JS 1691

Wei, YH 2390

Weichert, W 815

Weiderpass, E 1386, 1777

Weigelt, B 1531

Weigle, B 1034

Weinberg, D 26

Weiss, NS 146

Weiss, RA 2249

Weissbach, L 2305

Weisser, A 1893

Weitzen, R 773

Werneke, U 408

Wernli, M 1312

West, C 1796

West, CR 182, 253

Wester, K 2344

Whang-Peng, J 359

Wharton, CW 1450

Whitby, D 2145, 2181

White, SL 173

Wibault, P 55

Widegren, B 48

Wijnhoven, BPL 892

Wiley, KE 510

Wilkinson, C 1912

Willer, A 1893

Willers, H 1297

Williams, ED 2219

Williams, GT 1591

Wilsher, N 736

Wilson, H 2232

Wilson, WR 1084

Winkler, C 2305

Wirfält, E 122

Wishart, GC 1349

Wolf, G 815

Wolfe, MM 712

Wolk, A 2167

Wolowiec, D 2042

Wong, L-C 1747

Wong, W-L 1306

Wong, Z-W 20

Wood, LA 1885

Woods, LM 1367

Workman, P 2232

Wormhoudt, TAM 224

Wostl, W 1059

Wu, C-T 2378

Wu, C-W 1809

Wu, C-Y 1715

Wu, YT 2390

Wyke, SM 1850

Wykoff, CC 1235

Wyld, L 1486

Xia, X 1093

Xiao, A 761

Xie, L 1138

Xie, R 1508

Xie, Y 1636

Xu, J 510

Yacoub, GM 167

Yaegashi, N 2197

Yamada, A 1334

Yamada, M 1184

Yamagishi, H 665

Yamamoto, K 348

Yamamoto, N 87, 1125, 2092

Yamamoto, Y 236

Yamana, H 1334

Yamana, K 200

Yamauchi, S 1184

Yang, C-H 1715

Yang, J-Q 2349

Yang, L 2157

Yang, L-Y 2349

Yang, TL 1995

Yang, Y 2017

Yang, Z-L 2349

Yaniv, I 522

Yano, T 194

Yao, M 515, 712

Yashima, K 672

Yasugi, T 194

Yasumoto, K 436

Yatsuya, H 135

Yeh, K-H 1715

Yeo, W 1306

Yin, PH 2390

Yokomise, H 1555

Yokomori, T 1003

Yokoyama, Y 1627

Yoshida, H 414, 1600

Yoshida, T 348

Yoshikawa, J 1184

Yoshikawa, T 665

Yoshimura, N 1184

Yoshimura, T 135

Young, AR 874

Young, CYF 926

Young, MA 321

Yousef, GM 167

Yu, JC 1995

Yu, MC 1760

Yu, Q 476

Yuan, J-M 1756

Zahl, P-H 1686

Zaizov, R 522

Zajac, P 263

Zalutsky, MR 1469

Zaniboni, A 1521

Zänker, KS 230

Zappa, M 393, 1780, 1784

Zarkar, A 2106

Zeamari, S 1259

Zee, B 1306

Zeldis, J 955

Zeleniuch-Jacquotte, A 153

Zerp, S 917

Zhan, F 1814

Zhang, DH 1792

Zhang, S 1508

Zheng, SL 510

Zheng, W 2171

Zhong, S 1306

Zhou, W 712

Zhu, N 1457

Zhu, Y 1972

Zhu, Z 1863

Zietman, AL 578

Zimmermann, U 1230

Zorn, C 535

Zucchetto, A 2176

Zuetenhorst, JM 2073

Zunino, F 1464

Zwaan, RE 1144

Zwain, S 906

